# Effect of the Reassured Self-Compassion–Based School Program on Anxiety, Video Game Addiction, and Body Image Among Rural Female Adolescents: Retrospective Study

**DOI:** 10.2196/68840

**Published:** 2025-02-19

**Authors:** Areeg Zuair

**Affiliations:** 1 Community Health Nursing Department College of Nursing Taibah University Medina Saudi Arabia

**Keywords:** adolescents, rural, compassion-focused therapy, mental health, Saudi Arabia, school

## Abstract

**Background:**

The COVID-19 pandemic has amplified mental health challenges among adolescents, particularly in rural areas with limited access to services. In response, the Saudi government launched mental health campaigns and mandated schools to implement mental health programs. However, the effectiveness of these programs remains largely unreported.

**Objective:**

This study aims to determine the prevalence of anxiety disorder symptoms, video game addiction, and body image dissatisfaction, as well as to examine the effect of a school-based program, The Reassured Self, grounded in compassion-focused therapy, on anxiety symptoms, video game addiction, and body image dissatisfaction among rural adolescent females in Saudi Arabia.

**Methods:**

A retrospective secondary analysis of pre-post intervention data was used. All female middle school students (N=133; age: mean 13.7, SD 1.01 years) in grades 1-3 from a rural setting were included, with no exclusion criteria. Participants were recruited as part of a school-mandated mental health program. Data were collected at baseline (2 weeks before the intervention) and 2-3 weeks post intervention during school hours in a quiet classroom setting using teacher-supervised, printed surveys. Survey completion was voluntary, and students exhibiting high distress based on post data analysis were referred to the school health counselor for support. The intervention consisted of 3 sessions (30-35 minutes each) delivered over 2 weeks. Validated Arabic versions of the Spence Children's Anxiety Scale, Game Addiction Scale, and Body Image Discrepancy Assessment were used to measure outcomes 2 weeks post intervention (April to June 2024). Data were analyzed using linear mixed-effects regression models. Data were analyzed using linear mixed effects regression models.

**Results:**

Among the students, 25.8% (32/124) were identified as having high anxiety levels, with 19.3% (24/124) falling into the clinical concern or very high clinical concern categories. Additionally, 26% (32/123) were classified as problem gamers, while 14% (18/123) were categorized as addicted gamers. A smaller proportion, 5.1% (6/117), expressed a strong desire for thinness. Compared to pre-intervention levels, students exhibited significant reductions in anxiety symptoms (mean difference –4.11, 95% CI –6.98 to –1.24; *P*=.005) and video game addiction (mean difference –1.62, 95% CI –2.83 to –0.42; *P*=.009) following the program. However, changes in body image dissatisfaction were minimal and not statistically significant (mean difference 0.067, 95% CI –0.046 to 0.18; *P*=.057). High satisfaction scores, with a mean of 28.49 out of 35 (SD 6.31), indicated strong acceptability and cultural alignment of the intervention. High satisfaction scores indicated strong acceptability and cultural alignment with the intervention.

**Conclusions:**

The results highlight the potential for compassion-focused school programs to address mental health disparities in underserved adolescent populations. Future research should explore the long-term effects and scalability of such culturally adapted interventions.

## Introduction

The COVID-19 pandemic has profoundly impacted adolescent mental health worldwide, notably increasing anxiety disorder symptoms in this population [1-3]. With societies implementing lockdowns, enforcing social distancing, and facing unprecedented uncertainties, adolescents were exposed to substantial stressors that affected their psychological well-being [1-3]. A meta-analysis across 29 samples and 80,879 youth, conducted post the pandemic, highlighted a global increase in anxiety, with clinically significant symptoms nearly doubling, from 11.6% prepandemic to 20.5% post the pandemic [1]. This surge underscores the pandemic's enduring psychological effects on adolescents' mental health. Adolescence, a crucial developmental phase, often marks the onset of mental health disorders, with 50% emerging by age 14 and 75% by the age of 24 years [4]. Without timely intervention, these conditions can have long-lasting impacts on well-being and life trajectories.

Female adolescents have been disproportionately affected in the post–COVID-19 era, facing higher anxiety prevalence and greater symptom severity, necessitating targeted interventions that address their unique mental health needs [1,5]. Modifiable risk factors for anxiety disorders include game addiction and body image dissatisfaction, both of which significantly affect female adolescents [3,6]. Studies report an increase in gaming addiction during and after the pandemic, with prevalence estimates ranging between 2.3% and 29.4% [6]. Research shows a bidirectional relationship where excessive gaming escalates anxiety, and anxious adolescents use gaming as a coping mechanism [7]. Although many studies focus on male adolescents, emerging evidence indicates that female adolescents are similarly at risk for gaming addiction and associated mental health concerns [8,9]. This highlights the need for gender-specific research and interventions. Reports also indicate an increase in disordered eating and body image issues since the COVID-19 outbreak, partly due to increased exposure to appearance-focused social media and heightened internalization of social pressures [3,5].

In Saudi Arabia, the mental health landscape among adolescents is concerning, with 40.1% experiencing at least one lifetime disorder, 26.84% affected by anxiety, and 7.06% affected by eating disorders [10]. Female adolescents, particularly those in rural areas, are at higher risk for anxiety and body dissatisfaction, with a significantly higher anxiety prevalence than males [11]. For instance, 14.1% (424/3008) of Saudi females are at risk for generalized anxiety disorder, compared to 11.4% (342/3007) of males [11].

Rural female adolescents face additional barriers. Limited access to mental health services and cultural stigma exacerbate untreated mental health disorders, with only 0.5% of at-risk individuals in Saudi receiving treatment [11]. This calls for culturally appropriate, accessible interventions tailored to the specific needs of rural female adolescents in Saudi Arabia. Low-intensity mental health interventions that target anxiety, game addiction, and body image issues are critical to address these unmet needs.

Currently, a gap exists in the Saudi literature regarding school-based mental health and game addiction interventions for adolescents. Despite research on gaming addiction prevalence and related mental health issues, there is limited research on intervention programs, particularly those tailored to Saudi adolescents and female-specific needs [12]. Additionally, comprehensive programs that address both gaming addiction and associated mental health issues like anxiety are scarce, with most research focusing on treatment rather than prevention [8,12]. Also, our search failed to identify any mental health school program among Saudi adolescents. Additionally, there is a notable gap in research on the use of self-compassion interventions in populations outside of Western, educated, industrialized, rich, and democratic countries [13]. In Saudi Arabia, specifically, no reports were found on community- or school-based programs for female adolescents using any mental health intervention methods [10,12-16]. This study aims to fill this gap by providing valuable data on an underserved population: rural female adolescents, who often lack access to mental health services. Designed within the Saudi cultural context, this school-based intervention addresses interconnected issues of anxiety, game addiction, and body image dissatisfaction. The program emphasizes self-reassurance skills, empowering adolescents to manage their mental health independently—an approach particularly relevant in a cultural setting where mental health stigma may discourage individuals from seeking external help [15,17]. By fostering resilience, this intervention addresses critical mental health needs within this underserved population, contributing to the limited research in the area and offering insights that could inform future health policies and practices in Saudi Arabia.

To address these gaps, this study aimed to identify the prevalence of anxiety disorder symptoms, game addiction, and body image dissatisfaction among rural female adolescents; evaluate the efficacy of a compassion-focused therapy (CFT) school-based mental health intervention, The Reassured Self, in reducing anxiety disorder symptoms, game addiction, and body image dissatisfaction among rural female adolescents; and measure the acceptability of The Reassured Self intervention among rural female adolescents.

## Methods

### Design and Participants

This study used a retrospective design using existing data collected through a predesigned program implemented in a public school setting for quality improvement purposes (see Multimedia Appendix 1 for the checklist used to report the results of this retrospective database study, as recommended by the International Society for Pharmacoeconomics and Outcomes Research Task Force [18]). A pre-post design with no control group was used to evaluate the efficacy of a school-based mental health program, The Reassured Self. The intervention was conducted with middle school female students in an underserved rural area in the Medina region, Saudi Arabia. Saudi Arabian schools are required to offer health programs to students; however, reports on the efficacy and acceptability of these programs are limited. This study documents a partnership between a non-profit organization and a school to develop a mental health program and evaluates its efficacy and acceptability for future development.

Most rural areas in Saudi Arabia have a single girls' school. This project was implemented for all female middle school students (N=133) in grades 1-3, with no exclusion criteria, as the program was mandatory for all students (see Multimedia Appendix 2 for details on data completeness for intervention participants).

### Intervention Program: The Reassured Self

*The Reassured Self* is a culturally adapted, low-intensity intervention program designed for Saudi Arabian students. Based on the principles of self-compassion, the program integrates cultural and religious values with evidence-based practices to promote mental and emotional resilience. It addresses anxiety, game addiction, and body image dissatisfaction through tailored strategies. The program was delivered to middle school female students in a classroom environment, fostering peer support and shared learning experiences. Each class received three 30- to 35-minute sessions within 2 weeks. To ensure consistency, a trained nurse with experience on delivering the program to other schools delivered the same program to all classes. The intervention content focused on 5 core components, as outlined in Table 1.

**Table 1:** Detailed overview of the topics and descriptions covered in The Reassured Self intervention program^a^.

	Topic	Description
1	Foundational concepts of self-compassion	Self-blaming vs Self-reassurance: Introduces the difference between self-blame and self-compassion, guiding students to develop a supportive inner dialogue. Religious integration: Emphasizes Islamic principles written in the Quran and Hadith about compassion, assurance, self-blaming, and gratitude. Morning and evening supplications (Athkar) were incorporated for self-affirmation. Examples: “O reassured soul, return to your Lord well-pleased and pleasing to Him” (Surah Al-Fajr, 89:27-28) to emphasize fostering inner peace and self-assurance. “And I swear by the self-blaming soul” (Surah Al-Qiyamah, 75:2) to highlight self-reflection without destructive self-blame. Gratitude practices (Shukr): Students participated in gratitude journaling exercises, drawing on the verse: “If you are grateful, I will certainly give you more” (Surah Ibrahim, 14:7).
2	Building resilience	Challenge as growth: Teaches students to view challenges as opportunities for growth and resilience by using Quranic verses about patience, effort, and striving (Sabr and Sa’y). For example: “And that there is not for man except that [good] for which he strives” (Surah An-Najm, 53:39).
3	Addressing game addiction	Understanding dopamine and addiction: Explains how game addiction provides a temporary dopamine boost, leading to a false sense of achievement. Healthier coping strategies: Encourages real-life connections, exercises, and activities as healthier sources of satisfaction.
4	Reframing bullying	Understanding bullying behavior: Frames bullying as often stemming from the bully’s own self-blame, helping students empathize and avoid internalizing harmful behaviors. Responding with resilience: Guides students to respond to negativity with self-respect and compassion, fostering an empathetic school environment.
5	Practical applications and exercises	Self-compassion exercises: Includes activities like gratitude practices, physical self-care routines, and stress-management tools such as using stress-relief items. Exercise as self-care: Promotes physical exercise as a form of self-respect and care, enhancing both mental and physical well-being. Social skills for conflict resolution: Involves role-playing scenarios to provide practical guidance on maintaining self-respect in interactions with others, including handling criticism or bullying.

^a^The program integrates principles of self-compassion and Islamic values to foster emotional resilience, reduce anxiety, address game addiction, and promote healthy coping strategies. Each topic focuses on specific aspects of self-reassurance, resilience building, and practical exercises to enhance mental well-being in a culturally sensitive manner.

### Measurements and Data Collection

Data were collected at 2 time points: baseline (2 weeks before the intervention) and postintervention (2-3 weeks after the intervention). Assessments were conducted during school hours in a quiet classroom setting. The teacher distributed the printed surveys (paper-and-pencil format) to students, who were given 45 minutes to complete the questionnaires. The teacher remained available in the classroom to answer any questions. After the follow-up data collection, students exhibiting high levels of psychological distress, identified through their questionnaire responses, were referred to the school health counselor at that time for additional support.

#### Anxiety Symptoms

To assess clinically significant levels of anxiety symptoms, the Spence Children's Anxiety Scale (SCAS)–Arabic version was administered [19]. The SCAS is a 38-item questionnaire rated on a 4-point scale (0-3; never-always), with total scores reflecting the sum of responses [20]. This scale encompasses symptoms of the most prevalent *DSM-5* (*Diagnostic and Statistical Manual of Mental Disorders* [Fifth Edition]) anxiety disorders and is widely used in both clinical practice and research [21]. The SCAS includes 6 subscales that measure different dimensions of anxiety: separation anxiety, social phobia, obsessive-compulsive symptoms, panic or agoraphobia, physical injury fears, and generalized anxiety. Total anxiety was calculated by summing the scores from the individual subscales. In this study, the SCAS demonstrated excellent internal consistency (α=0.94). *t*-Scores and cutoffs for the SCAS are available on the SCAS website and are categorized by age and gender [22]. For girls aged 12-15 years, a total score cutoff of ≥60 (84th percentile) suggests clinically significant anxiety symptoms.

#### Video Game Addiction

A newly modified Arabic translation of the 7-item version of the Game Addiction Scale (GAS) was used to measure video game addiction [23]. Responses were given on a 5-point scale (1=“never” to 5=“very often”), with a total score range of 7-35. The original Arabic version of the GAS served as the basis for this translation [24]. In this study, the GAS-Arabic demonstrated good internal consistency (α=0.81).

#### Video Game Addiction Status

To classify participants based on their level of video game engagement, responses to the core 4-item criteria for addiction (withdrawal, relapse, conflict, and problems) were evaluated according to methods recommended in recent literature [25]. Participants were divided into 3 groups: addicted gamers, problem gamers, and engaged gamers. Respondents who scored 3 or higher on all 4 items were classified as Addicted Gamers. Those who scored 3 or higher on 2 or 3 of the 4 items were categorized as problem gamers, indicating some signs of problematic gaming behavior. Respondents who did not meet the criteria for either addicted or problem gamers were classified as engaged gamers, reflecting low engagement without signs of problematic behavior.

#### Body Image Dissatisfaction

Body image dissatisfaction was assessed using a series of silhouettes representing 4 different body sizes [26]. Students selected the silhouette that best represented their ideal body size and the one that most resembled their current body size. The difference between these selections (ideal vs current) indicated the level of body image discrepancy, with scores ranging from –3 to +3. A negative score suggested a desire for thinness (preference for a smaller body size), while a positive score indicated a desire for fullness (preference for a larger body size) [27]. Participants were categorized as follows: high drive for thinness (scores less than –1.5), low drive for thinness (scores between –1.5 and 0), and no drive for thinness (scores of 0 or higher).

#### Program Reaction

Participant feedback was collected using the Kirkpatrick model's level 1 evaluation 2-3 weeks post the program. The level 1 evaluation, also known as Reaction, assesses participants' immediate responses to a training or intervention [28]. It captures how participants felt about the experience, evaluating satisfaction, perceived ease and clarity, engagement (perceived effectiveness), and perceived relevance of the content [28]. A 7-item Likert scale was used, with responses ranging from 1 (totally disagree) to 5 (totally agree), resulting in a total score range of 7-35.

### Statistical Analysis

#### Overview

Descriptive statistics (means, SDs, and frequencies) were calculated for all variables. A series of linear mixed models were used to examine the effects of the intervention on 3 dependent variables—anxiety symptoms, video game addiction, and body image discrepancy—measured at 2 time points (before and post the intervention). The models included time as a fixed effect and student ID as a random effect to account for within-subject variability across repeated measures. Restricted maximum likelihood estimation was used to fit the models. Grade levels were tested as fixed effects, but neither demonstrated significant associations with the outcomes or interactions with time. Consequently, these variables were excluded from the model. Pairwise comparisons of the estimated marginal means were conducted using least significant difference adjustment. The assumption of homoscedasticity (constant variance) appeared mostly satisfied, and the residuals were approximately normally distributed. Linearity between time and each dependent variable was reasonable, as no strong nonlinear patterns were observed. Also, correlation analyses were conducted to identify potential confounding variables, including age, baseline anxiety, body image discrepancy, and gaming addiction.

Participants with valid data on anxiety symptoms, video game addiction, or body image discrepancy at either baseline or follow-up were included in the analysis. Linear mixed models use all available data, regardless of missing outcome data, under the assumption that data are missing at random. Data were found to be missing completely at random, as indicated by Little's MCAR test (*χ*²_135_=110.5, *P*=.94). No cases were missing more than 75% of data, which is the recommended threshold for analyzing the SCAS.

#### Power Analysis

A post hoc power analysis was conducted using G*Power (version 3.1.9.7) to evaluate the achieved power for detecting changes in anxiety levels, game addiction, and body image discrepancy across pre- and postintervention time points. The paired samples *t* test was selected as the appropriate statistical test for the pre-post design, focusing on differences between dependent means. Effect sizes (dz) were calculated as 0.25, 0.24, and 0.076 based on the observed mean differences and SDs of the difference scores for anxiety, game addiction, and body image discrepancy, respectively.

The analysis was performed with an alpha level of 0.05 and total sample sizes of 133, 132, and 130 participants for anxiety, game addiction, and body image discrepancy, respectively. The results indicated that the study had an 89% and 86% probability of detecting a true effect for anxiety and game addiction, respectively. However, the post hoc power for body image discrepancy was approximately 13.8%, indicating that the study had very low power to detect such a small effect. This suggests that the study may not have been adequately powered to identify significant changes in body image discrepancy.

### Ethical Considerations

Given the retrospective nature of the study, all students participated in the program as it was mandated by the school health unit as part of the standard curriculum. Written consent was obtained from students specifically for their participation in completing the surveys associated with the program. Information about the surveys, including their purpose and voluntary nature, was communicated to the students in an age-appropriate manner. This included verbal explanations by teachers and written summaries that were easy to understand. Students were assured that completing the surveys was voluntary and would not affect their grades or school standing. To encourage participation, all students received a small gift (a relaxation squeeze toy) after completing the program satisfaction survey. Parental consent was not required, as the program itself was a required school activity, and the decision to waive parental consent for the surveys followed ethical guidelines for school-based health programs. This approach was approved by the University of Taibah Nursing College Ethical Review Committee (IRB No. TUCN-REC 11 2024). To ensure the privacy and confidentiality of participants, all data from school project records were securely stored and deidentified prior to analysis.

## Results

### Students’ Characteristics

Table 2 presents the descriptive statistics for the study variables, including age, anxiety symptoms, video game addiction, and body image discrepancy. A total of 133 students participated in the program. However, only 93% (124/133) completed the preanxiety symptoms survey, 92% (123/133) completed the video game addiction, and 88% (117/133) completed the body image discrepancy assessment (see Multimedia Appendix 2 for details on data completeness for intervention participants). The mean age of participants was 13.72 (SD 1.01) years. Total anxiety symptoms had a mean score of 47.60 (SD 22.02), indicating a moderate level of anxiety across the sample. Among the anxiety subscales, panic or agoraphobia symptoms were the highest, with a mean of 9.38 (SD 6.76), followed by obsessive-compulsive symptoms (mean 8.79, SD 4.54) and generalized anxiety symptoms (mean 8.66, SD 4.55). Physical injury fears (mean 7.64, SD 3.75) and separation anxiety (mean 6.31, SD 3.88) were also prominent. Social phobia symptoms had a mean of 6.82 (SD 4.12), indicating a relatively lower prevalence compared to other anxiety types.

**Table 2:** Descriptive statistics for age, anxiety symptoms, video game addiction, and body image discrepancy among rural adolescent female students^a^.

Descriptive statistics		Mean (SD) values
Age (years; n=133)		13.72 (1.01)
**Total anxiety symptoms (n=124)**		47.60 (22.02)
	Generalized anxiety symptoms	8.66 (4.56)
	Physical injury fears symptoms	7.64 (3.75)
	Separation anxiety symptoms	6.31 (3.88)
	Panic agoraphobia symptoms	9.38 (6.76)
	Social phobia symptoms	6.82 (4.12)
	Obsessive-compulsive symptoms	8.79 (4.54)
Video game addiction (n=123)		15.57 (6.67)
Body image discrepancy (n=117)		–0.197 (0.87)

^a^Descriptive statistics summarizing the mean and SD for age, anxiety symptoms, video game addiction, and body image discrepancy among rural adolescent female students who participated in The Reassured Self intervention program.

The mean score for video game addiction was 15.57 (SD 6.67), indicating a moderate level of engagement or potential addiction to gaming. The body image discrepancy score had a mean of –0.20 (SD 0.87), suggesting a slight drive for thinness among the sample, with negative values indicating a preference for a thinner body size compared to current perception. Correlations between baseline variables showed a significant, moderate positive relationship between anxiety and gaming addiction (r=0.457, *P*<.001). Age and body image discrepancy demonstrated weak, nonsignificant correlations with other variables.

### Characterizing Mental Health Status

The distribution of anxiety symptoms among students reveals that a substantial portion experience elevated or clinically concerning levels across various anxiety types (Table 3). Specifically, 25.8% (32/124) of students had a high anxiety level, and 19.3% (24/124) fall within the clinical concern or very high clinical concern category, indicating that a significant subset may require attention. Generalized anxiety is particularly prevalent, with 33.1% (41/124) of students classified in the clinical concern or very high clinical concern categories. In addition, fears related to physical injury show one of the most severe patterns, with 33.9% (42/124) of students in the very high clinical concern category, highlighting this as a critical area for mental health support. Separation anxiety also shows a high prevalence, with 33.9% (42/124) of students in the clinical or very high clinical concern categories. Symptoms of panic and agoraphobia are notable as well, with 28.2% (35/124) of students in the very high clinical concern category, indicating a significant impact. Obsessive-compulsive symptoms present another area of concern, with 21% (26/124) of students in the very high clinical concern category. In contrast, social phobia has a lower prevalence of severe concern, with only 2.4% (3/124) of students in the very high clinical concern category.

**Table 3:** Frequency and percentage distribution of anxiety levels, video game status, and body image dissatisfaction among rural adolescent female students.

Anxiety levels (N=124)^a^	Normal range	Elevated anxiety	Clinical concern	Very high clinical concern
Anxiety symptoms, n (%)	92 (74.2)	8 (6.5)	5 (4)	19 (15.3)
Generalized anxiety, n (%)	61 (49.2)	22 (17.7)	25 (20.2)	16 (12.9)
Physical injury fears, n (%)	38 (30.6)	20 (16.1)	24 (19.4)	42 (33.9)
Separation anxiety, n (%)	55 (44.4)	27 (21.8)	16 (12.9)	26 (21)
Panic and agoraphobia, n (%)	54 (43.5)	18 (14.5)	17 (13.7)	35 (28.2)
Social phobia, n (%)	93 (75)	12 (9.7)	16 (12.9)	3 (2.4)
Obsessive-compulsive, n (%)	38 (30.6)	30 (24.2)	30 (24.2)	26 (21)
Video game status (N=123)^a^, n (%)	Engaged gamers: 73 (59)	Problem gamers: 32 (26)	Addicted gamers: 18 (14)	Addicted gamers: 18 (14)
Body image dissatisfaction (N=117)^a^, n (%)	No drive for thinness: 77 (65.8)	Low drive for thinness: 34 (29.1)	High drive for thinness: 6 (5.1)	High drive for thinness: 6 (5.1)

^a^Total number of completed surveys.

Overall, the findings suggest that while some anxiety symptoms present moderate concern, specific areas—particularly physical injury fears, separation anxiety, and obsessive-compulsive symptoms—are more prevalent at clinically concerning levels, underscoring the need for focused mental health support in these domains.

Regarding body image dissatisfaction, the results indicate a slight drive for thinness among most of the students. Approximately 29.1% (34/117) of students exhibit a moderate inclination toward weight reduction, while a smaller proportion, 5.1% (6/117), express a strong desire for thinness.

### Characterizing Video Game Status

The data on video game status among students reveals varying levels of gaming involvement, with a significant portion showing signs of problematic or addictive behavior (Table 3). Overall, 59% (73/123) of the students are categorized as engaged gamers, indicating regular gaming without notable adverse effects. However, 26% (32/123) are identified as problem gamers, suggesting a level of gaming that may interfere with daily responsibilities or personal well-being. Additionally, 14% (18/123) of students fall into the addicted gamers category, indicating a more severe level of dependency that likely impacts various aspects of their lives.

### Effectiveness of The Reassured Self Program

The Reassured Self intervention demonstrated a positive effect in reducing anxiety levels among students, as shown by a significant decrease in anxiety scores from preintervention to follow-up (see Table 4). The effect size, with a mean difference of –4.11, was small but statistically significant (*P*=.005). Additionally, the intervention had a beneficial impact on reducing video game addiction, with an estimated mean reduction of –1.62 in game addiction scores, which was also statistically significant (*P*=.009). In contrast, although the intervention led to a slight decrease in the drive for thinness, this change was not statistically significant (*P*=.057), suggesting a minimal effect on body image dissatisfaction. Overall, these findings indicate that the intervention was effective in reducing both anxiety and game addiction among students, while its impact on body image discrepancy remains inconclusive.

**Table 4:** Changes in anxiety symptoms, video game addiction, and body image dissatisfaction from baseline to follow-up^a^.

Outcome	Baseline, mean (SE) [95% CI]	Follow-up, mean (SE) [95% CI]	Within group differences change from follow-up–baseline	
			Mean difference [95% CI]	*P* value
Anxiety symptoms (n=133)	47.137 (1.93) [43.03 to 50.95]	43.32 (2.13) [38.81 to 47.25]	–4.11 [–6.98 to –1.24]	.005
Video game addiction (n=132)	15.45 (0.59) [14.28 to 16.63]	13.83 (0.61) [12.63 to 15.03]	–1.62 [–2.83 to –0.42]	.009
Body image dissatisfaction (n=130)	–0.19 (0.08) [–0.35 to 0.038]	–0.125 (0.07) [–0.27 to 0.02]	0.067 [–0.046 to 0.18]	.057

^a^This table presents the results of linear mixed-effects models, which accounted for repeated measures within individuals, to evaluate changes in anxiety symptoms, video game addiction, and body image dissatisfaction from baseline to follow-up among participants of The Reassured Self intervention program. Adjustments for multiple comparisons were made using the least significant difference method.

### Program Reaction

The program received positive feedback across multiple dimensions, as reflected in the high mean scores for various aspects of participant satisfaction (Table 5). The overall satisfaction sum score ranged from 7 to 35, with a mean of 28.49 (SD 6.31), indicating a generally favorable response. The overall results suggest that participants responded positively to the program, finding it engaging, clear, and psychologically beneficial, with a strong likelihood to recommend it to others.

**Table 5:** Descriptive statistics for reaction measures of The Reassured Self program (N=123)^a^.

Measure	Mean (SD) values
Overall satisfaction score	28.49 (6.31)
Perceived ease and clarity	4.41 (0.88)
Enjoyment and satisfaction	4.16 (1.14)
Psychological benefits gained	4.14 (1.11)
Program relevance and value	3.9 (1.29)
Teaching effectiveness	3.97 (1.13)
Engagement and attention retention	4.15 (1.09)
Likelihood of recommending the program	4.2 (1.20)

^a^This table presents the mean scores and SDs for various aspects of participant satisfaction with The Reassured Self program. The consistently high scores across all measures reflect positive feedback and strong acceptability of the intervention.

## Discussion

### Principal Findings

This study conducted a retrospective analysis of data collected from female adolescents who participated in a CFT-based school program, The Reassured Self, in a rural setting. The program, collaboratively implemented by a nonprofit organization and an academic institution, aimed to reduce anxiety, video game addiction, and body image dissatisfaction among rural Saudi female adolescents, addressing critical mental health challenges within this population. The findings revealed a high prevalence of anxiety and video game addiction, consistent with national averages. The intervention effectively reduced anxiety and video game addiction symptoms but showed no improvement in body image dissatisfaction, highlighting its potential as a health education tool.

### Anxiety Reduction

The overall high anxiety symptom prevalence of 25.8% (32/124) and the high severe anxiety symptoms of 19.3% (24/124) in our sample are comparable to global postpandemic figures (20.5%) [1] and align closely with the recent national prevalence of lifetime anxiety disorders among adolescents (26.84%) [10]. Our results underscore the urgent need for interventions targeting adolescent anxiety in underserved areas. Recent literature argues that school well-being programs should consider children’s and adolescents’ anxiety levels as a key metric for measuring program impact [29]. The Reassured Self program demonstrated a significant reduction in anxiety symptoms, suggesting that a culturally adapted CFT approach can effectively address anxiety. This aligns with recent meta-analyses and systematic reviews, which consistently show that compassion-based interventions reduce anxiety and psychological distress among adolescents [30-32].

This study’s findings also align with recent evidence on compassion-based school interventions. For instance, Maratos et al [29] evaluated a Compassionate Mind Training (CMT-Pupils) intervention among Year 7 students aged 11-12 and found significant improvements in anxiety levels compared to the control group. Similar to our intervention, CMT-Pupils emphasized emotional regulation and prosocial behaviors through compassion-based practices, including mindfulness and self-compassion. Their study highlighted the importance of promoting kindness, emotional awareness, and classroom inclusivity, which aligns with the core mechanisms of The Reassured Self program.

Further supporting the importance of self-compassion in adolescent mental health, Hammad et al [16] conducted a cross-sectional study in Saudi Arabia that explored the relationship between perceived stress and self-compassion among adolescents during the COVID-19 pandemic. Their findings revealed a significant negative correlation (r=–0.460, *P*<.001) between perceived stress and self-compassion levels, suggesting that higher self-compassion is associated with lower levels of stress. These results underscore the role of self-compassion as a protective factor for adolescent mental health, reinforcing the theoretical foundation of The Reassured Self program, which aims to reduce anxiety by fostering self-compassion and emotional regulation. CFT is rooted in evolutionary psychology and attachment theory, incorporating mindfulness through activities designed to foster self-compassion toward oneself and others, particularly in relation to self-criticism, self-blame, and shame [17,31,33]. Recommended techniques and exercises to develop self-compassion include self-talk and understanding the evolved nature of the human mind, along with the origins of self-criticism and self-blame [17]. Therefore, the core focus of The Reassured Self program is to introduce students to the concepts of self-blaming and self-reassurance, fostering a supportive inner dialogue and emotional regulation.

However, while The Reassured Self program demonstrated reductions in anxiety, our study did not directly measure emotional regulation or self-compassion processes—key mechanisms observed in Petrocchi et al [33] and other self-compassion studies. Tools such as the Forms of Self-Criticizing/Attacking and Self-Reassuring Scale, developed by Gilbert et al [34] (the founder of CFT), provide validated measures for assessing self-criticism and self-reassurance. Incorporating these tools in future studies would enable a more comprehensive evaluation of how The Reassured Self program works. Specifically, measuring changes in emotional regulation, self-reassurance, and self-criticism could elucidate the mechanisms driving the reduction in anxiety and gaming addiction. As literature suggests, a decrease in self-criticism and self-blame is strongly associated with a reduction in anxiety [17,31,33]. Including these intermediate outcomes will help validate the program's effectiveness and provide deeper insights into its psychological processes. Overall, our findings suggest that culturally relevant, compassion-based programs can provide a viable solution for reducing anxiety in rural, underserved contexts. By addressing both the reduction of anxiety symptoms and fostering emotional regulation, such interventions hold promise as scalable and effective mental health solutions for adolescents. Also, our approach is supported by recent findings that advocate for brief, targeted interventions to effectively engage adolescents, particularly in an era where social media usage has impacted attention spans [35]. A recent meta-analysis of brief school mental health interventions found that programs lasting fewer than 4 sessions or 250 minutes resulted in significant, albeit small, improvements in students' mental health when assessed 1 month, 6 months, and 1 year post intervention [35].

### Reduction in Video Game Addiction

The high baseline prevalence of video game addiction (14.6%, 18/123) among rural female adolescents in our study underscores the need for targeted mental health interventions in this area. This prevalence is close to global averages, which show approximately 12% of adolescents are at risk for problematic gaming, highlighting the specific challenges faced by this population [36]. However, the prevalence in our study is lower than that in the recent national report for the Madinah region (28%), and the overall national prevalence of 20.45% (897/4431) [37].

The program’s focus on self-compassion and emotional resilience appears to have positively impacted gaming behaviors, as evidenced by a significant decrease in video game addiction scores. Furthermore, the significant correlation between anxiety and gaming addiction underscores their interconnectedness, aligning with existing research that highlights gaming as a coping mechanism for anxiety [7].

This finding supports research suggesting that interventions emphasizing emotional regulation and coping skills such as exercise can reduce problematic gaming behaviors [38]. For example, a recent study investigating the relationship between internet gaming disorder, sleep quality, self-compassion, physical activity, and psychological distress among young adults aged 18-24 years found that internet gaming disorder was positively associated with interpersonal stress and depressive symptoms. Additionally, self-compassion and exercise behavior were found to mediate the relationship between depressive symptoms and sleep quality [39].

By providing alternative coping mechanisms, The Reassured Self may help reduce adolescents' reliance on gaming to manage emotional distress, addressing a critical mental health concern in this demographic. However, as discussed earlier, the lack of measurement of changes in emotional regulation, or adapting exercise behaviors, which could be assessed using validated tools, limits the conclusions that can be drawn about the program's overall efficacy.

### Body Image Dissatisfaction

The notable prevalence of body image dissatisfaction, with 5.1% (6/117) of students showing a strong drive for thinness, reveals another area for potential intervention. Although the program’s impact on body image dissatisfaction was minimal and nonsignificant, the slight reduction in drive for thinness suggests that longer or more targeted interventions may more effectively address body image issues. Body image is deeply influenced by social and cultural factors, and this program provides a foundation for future interventions to build upon by incorporating more specific strategies to address body image concerns in rural contexts. While CFT has shown success in improving body image in other studies [30], the short duration of our program may have limited its impact on this complex issue. Additionally, this study found an average of 1 size discrepancy among the students, aligning with other research indicating that rural students may have low body image discrepancy [40,41]. Future iterations of the program may need to incorporate more targeted components to address body image issues specific to the cultural context.

### Program Reaction

The integration of Islamic values and culturally sensitive practices, such as gratitude and self-compassion, likely enhanced the intervention's acceptability and effectiveness. Student feedback indicated high levels of satisfaction regarding the program's ease and clarity, perceived effectiveness, and content relevance. The concept of self-compassion, rooted in Islamic culture and religious practice, fosters self-reassurance and reduces self-blame [16]. Cultural and contextual considerations played a crucial role in the program’s design and implementation, which aligned with current recommendations for culturally adapting mental health interventions through self-compassion [17]. In addition, the program's success in a rural setting addresses a critical gap in mental health services for underserved populations and supports the need for global mental health interventions [42,43].

### Limitations and Recommendations

This study’s findings have important implications for designing school-based mental health programs in rural or underserved areas. To ensure the effective delivery and sustainability of such interventions, several strategies should be prioritized: (1) training mental health professionals, educators, and community facilitators is critical for delivering the intervention effectively. (2) Partnerships with local organizations and schools can enhance resource availability and facilitate recruitment in resource-limited settings. (3) Leveraging community partnerships: the collaboration demonstrated in this study—between a nonprofit organization, an academic institution, and a local school—provides a viable model for delivering mental health interventions in underserved areas. This approach aligns with broader recommendations to leverage community partnerships to enhance mental health support in schools [43,44]. (4) Advocacy efforts should focus on investments in culturally adapted, school-based mental health interventions to ensure they can reach underserved populations and address mental health disparities in context-specific and meaningful ways. (5) Future interventions should explore digital education methods, such as webinars, web-based workshops, and gaming-based mental health curricula, to enhance accessibility and engagement, particularly among rural students [45,46]. (6) Future research should investigate the intervention's long-term effects, including its impact on academic performance (eg, grades, school engagement) and social functioning (eg, relationships with peers, family members, and teachers). Understanding whether mental health improvements translate into better academic and social outcomes would provide a more holistic evaluation of the program’s effectiveness. (7) Further exploration of specific cultural factors, such as family dynamics, social stigma, and rural-urban differences, is necessary to understand their contributions to the program's effectiveness. Adapting the intervention for broader cultural and socioeconomic contexts is critical for expanding its applicability.

However, several limitations should be considered when interpreting these results. The retrospective design and lack of a control group limit causal inferences. Additionally, the short follow-up period (2-3 weeks) may not capture long-term effects or potential relapses [17]. The specific demographic focus on rural Saudi female adolescents may also limit generalizability to other populations. To address these limitations and build on this study, future research should (1) incorporate randomized controlled trial designs with active control groups to establish causality; (2) extend follow-up periods to evaluate the sustainability of intervention benefits over time; (3) compare The Reassured Self program with other evidence-based approaches, such as cognitive-behavioral therapy and mindfulness-based stress reduction, to assess relative effectiveness and adaptability; (4) examine the specific components of the program and their relative contributions to outcomes to refine and optimize the intervention; and (5) investigate the interplay between cultural factors and program outcomes to ensure effective adaptations for different cultural and socioeconomic contexts.

### Conclusions

Adolescents in rural Saudi Arabia face significant mental health challenges, with high rates of anxiety, video game addiction, and, to a lesser extent, body image dissatisfaction. This study's findings underscore the potential of a culturally adapted CFT program, The Reassured Self, to address these issues. Collaboratively implemented by a non-profit organization and an academic institution, the program successfully reduced anxiety symptoms and problematic gaming behaviors among rural female adolescents, providing a promising model for school-based mental health interventions in underserved areas.

The integration of Islamic values and culturally sensitive practices, such as gratitude and self-compassion, likely enhanced the program’s acceptability and relevance, as reflected in the high levels of student satisfaction with the intervention's content and delivery. The Reassured Self demonstrates the feasibility and effectiveness of a culturally sensitive, compassion-focused intervention for adolescent mental health in rural Saudi Arabia. The promising findings of The Reassured Self program highlight its potential for widespread implementation in schools and communities, particularly in underserved areas. By addressing the unique challenges faced by this underserved population, the program contributes valuable insights for global mental health initiatives aimed at reducing disparities in mental health access and support. Future efforts to refine and scale the intervention can inform broader applications in diverse cultural and socioeconomic contexts.

## Appendix

Multimedia Appendix 1 Checklist for Reporting Results of Retrospective Database Studies from the ISPOR Task Force.

Multimedia Appendix 2 Data Completeness for Intervention Participants.

## Abbreviations

CFT: compassion-focused therapy
CMT-Pupils: Compassionate Mind Training
DSM-5: Diagnostic and Statistical Manual of Mental Disorders (Fifth Edition)
GAS: Game Addiction Scale
SCAS: Spence Children's Anxiety Scale

## Data Availability

The datasets generated and analyzed during this study are available from the corresponding author on reasonable request.
