# Supplementary material for: Effect of the Reassured Self-Compassion–Based School Program on Anxiety, Video Game Addiction, and Body Image Among Rural Female Adolescents: Retrospective Study
Source: JMIR Form Res. 2025 Feb 19;9:e68840. doi: 10.2196/68840 (PMC11888012; doi:10.2196/68840)
Supplement: Multimedia Appendix 1 [file formative_v9i1e68840_app1.pdf]

Table 1. Checklist for Reporting Results of Retrospective Database Studies from the ISPOR Task Force:

	Section	Checklist Item	Key Considerations	Result
**1**	Data Sources	Relevance	Were the attributes of the database sufficiently described (e.g., population characteristics, benefit design)?	Adequate
		Reliability and Validity	Were data quality checks performed (e.g., missing values, duplicate records, outliers)?	Adequate
		Data Linkages	Were linkages across sources or care sites appropriate, addressing coding/reporting differences?	N/A
		Eligibility Determination	Was eligibility accurately determined, and were flawed approaches (e.g., based on single claims) avoided?	Adequate
**2**	Study Design	Data Analysis Plan	Was a data analysis plan, including hypotheses, developed a priori?	Adequate
		Design Selection	Was the chosen design justified (e.g., cohort, case-control) with strengths and limitations addressed?	Adequate, but limitations noted
		Research Design Limitations	Were biases (e.g., selection, maturation, regression to the mean) identified and addressed?	Adequate
		Treatment Effect	Was a comparison group included? Was the process for identifying the group and its similarity to the treatment group described?	Limitation
**3**	Study Population	Sample Selection	Were inclusion/exclusion criteria, along with the steps to derive the final sample, explicitly described?	Adequate
		Censoring	Was censoring (e.g., eligibility duration) addressed, and its impact on findings discussed?	Adequate
		Operational Definitions	Were case definitions and endpoints clearly defined (e.g., using diagnosis codes)?	Adequate
		Definition Validity	Was a rationale for definitions provided, with sensitivity analyses for controversial or uncertain definitions?	Adequate
		Timing of Outcomes	Was the temporal relationship between exposure and outcome clear?	Partial
		Event Capture	Could the data reliably identify interventions and outcomes (e.g., services not captured in claims data)?	Adequate
		Disease History	Did the analysis consider the natural history of the disease (e.g., long follow-up for chronic conditions)?	N/A

**4**	Resource Valuation	Resource Identification	Were all resources (e.g., costs) affected by the intervention defined and measured?	N/A
		Pricing Adjustments	Were resource prices consistent with the study's perspective (e.g., insurer, patient, society) and adjusted for inflation/geography?	N/A
**5**	Statistical Analysis	Control Variables	Were methods to address confounding (e.g., stratification, multivariate techniques) described?	Adequate
		Statistical Model	Was the rationale for the chosen statistical model explained, including handling of skewed data and hierarchical data structures?	Adequate
		Sensitivity to Influential Cases	Were sensitivity analyses performed to evaluate the impact of influential cases or outliers?	Adequate
		Relevant Variables	Were all hypothesized variables included in the model?	Adequate
		Testing Assumptions	Were statistical assumptions tested (e.g., regression diagnostics, omitted variables)?	Adequate
		Multiple Testing	Were adjustments made for multiple comparisons to avoid spurious findings?	Adequate
		Model Prediction	Did the authors discuss the model's predictive validity (e.g., goodness-of-fit, split-sample validation)?	Partial
**6**	Discussion	Causality	Did the authors provide plausible explanations for findings and rule out alternative causes?	Adequate, with limitations noted
		Practical Significance	Were statistical findings interpreted in terms of clinical or economic relevance?	Adequate
		Generalizability	Were the populations and settings to which the results can be generalized clearly discussed?	Adequate
