# Supplementary material for: Effect of the Reassured Self-Compassion–Based School Program on Anxiety, Video Game Addiction, and Body Image Among Rural Female Adolescents: Retrospective Study
Source: JMIR Form Res. 2025 Feb 19;9:e68840. doi: 10.2196/68840 (PMC11888012; doi:10.2196/68840)
Supplement: Multimedia Appendix 2 [file formative_v9i1e68840_app2.pdf]

Data Completeness for Intervention Participants

Scale/Variable	Total Intervention Participants	Pre-Intervention Data Provided	Post-Intervention Data Provided	No Pre/Post Data	LMM sample size	Notes
Anxiety Symptoms	133	124	123	0	133	All students contributed either pre or post data.
Game Addiction	133	123	123	1	132	One student had no pre- or post-data.
Body Image Discrepancy	133	117	120	3	130	Three students had no pre- or post-data.

Notes:

All 133 students attended the intervention.

No students had completely missing data across all scales.
